# Marked Improvement in Treatment-Intolerant Obsessive-Compulsive Disorder in an Adolescent Using a Glutamatergic Regimen With Dextromethorphan, Piracetam, and Goldenseal Root

**DOI:** 10.7759/cureus.104617

**Published:** 2026-03-03

**Authors:** Ngo Cheung

**Affiliations:** 1 Psychiatry, Independent Research, Hong Kong, HKG

**Keywords:** ampa receptor, glutamatergic modulation, herbal drugs, nmda receptor antagonist, obsessive-compulsive disorder (ocd), ocd/anxiety disorders

## Abstract

A 16-year-old boy with severe obsessive-compulsive disorder (OCD), characterized by health-focused obsessions, intrusive ruminations, derealization, and significant functional impairment, failed to tolerate standard serotonergic agents (escitalopram, fluoxetine) and bupropion due to worsening anxiety, somatic concerns, or headaches. An oral glutamatergic regimen was initiated with dextromethorphan 30 mg/day for N-methyl-D-aspartate (NMDA) antagonism, piracetam 1,200 mg/day for AMPA modulation, and goldenseal (Hydrastis canadensis) root extract 600 mg/day as a botanical CYP2D6 inhibitor to prolong dextromethorphan exposure, alongside low-dose pregabalin, risperidone, and aripiprazole for ancillary support. Within four weeks of adding goldenseal, symptoms markedly improved, meeting remission thresholds: the Patient Health Questionnaire-9 (PHQ-9) score fell to 2, GAD-7 score to 3, obsessive thoughts and derealization resolved, headaches ceased, and full school attendance and functioning returned. These benefits lasted for two months, and there were no negative effects from goldenseal. This case indicates that a cost-effective, entirely oral amalgamation of a prevalent cough suppressant, a nootropic, and a botanical supplement may yield ketamine-like glutamatergic advantages for patients with OCD who are intolerant to conventional treatments; however, controlled studies are required to validate efficacy and safety.

## Introduction

Obsessive-compulsive disorder (OCD) emerges in roughly 1-3% of young people and commonly begins before adulthood, disrupting school performance, friendships, and family life [1]. Core symptoms--unwanted obsessions and repetitive compulsions--often coexist with anxiety, low mood, or dissociative complaints, adding to the clinical burden and making care more complex [2,3]. The objective of this case report is to describe the clinical and safety outcomes of an oral glutamatergic regimen in an adolescent with treatment-intolerant OCD.

Standard practice combines exposure-based cognitive-behavioral therapy with a selective serotonin reuptake inhibitor (SSRI). Even so, only about half to two-thirds of children and adolescents achieve meaningful relief, and bothersome adverse effects, such as activation, weight gain, or emotional flattening, can limit adherence [4]. When first-line treatment stalls, clinicians frequently add low-dose antipsychotics, but evidence is limited, and safety concerns remain [5].

Increasing attention has shifted toward glutamatergic signalling in the cortico-striato-thalamo-cortical loop, a circuit repeatedly implicated in OCD pathophysiology [6,7]. Agents that dampen or modulate glutamate transmission, therefore, represent a promising alternative. Intravenous ketamine, a non-competitive N-methyl-D-aspartate (NMDA) receptor antagonist, can cut obsessional distress within hours, though benefits are usually brief and infusion logistics limit routine use [8,9].

Intravenous ketamine has put glutamatergic modulation on the psychiatric map, yet its cost, monitoring requirements, and abuse potential make it hard to scale. Orally delivered agents could fill that gap. Dextromethorphan (DXM) is a well-known, over-the-counter NMDA antagonist that recently entered mainstream practice when combined with bupropion in the fixed-dose product Auvelity. The bupropion component inhibits cytochrome P450 2D6 (CYP2D6), slowing the metabolism of DXM and producing clinically meaningful antidepressant and anxiolytic effects [10]. Early reports, including an ongoing controlled trial, hint that the same strategy might reduce obsessive-compulsive symptoms as well [11,12].

Building on this idea, Cheung proposed a fully oral, low-cost "ketamine-mimic" stack: DXM for NMDA blockade; a strong CYP2D6 inhibitor to keep DXM levels high; piracetam to up-regulate AMPA throughput; and optional glutamine to replenish presynaptic pools while buffering against excitotoxicity [13]. When prescription inhibitors such as bupropion or fluoxetine are poorly tolerated, botanicals that contain CYP2D6-blocking alkaloids, notably goldenseal (Hydrastis canadensis) with its berberine and hydrastine content, have been suggested as substitutes, though batch variability and limited safety data remain significant concerns [14,15].

Here, we present the first detailed account of an adolescent with treatment-intolerant OCD who used a goldenseal-supported DXM regimen after failing, or being unable to tolerate, standard serotonin-based options. The case illustrates a pragmatic, outpatient route for exploring glutamatergic interventions without the infrastructure of an infusion suite.

## Case presentation

The patient was a 16-year-old boy who first attended the clinic complaining of uncontrollable worries about his health, intrusive thoughts, and trouble concentrating as he began Form 4 in Hong Kong. He fixated on two minor head injuries--one sutured in 2018 and another domestic knock several years earlier--and spent hours online searching for possible neurological consequences, which in turn heightened his anxiety. He also reported avolition, poor sleep, and restlessness. His mother confirmed long-standing academic pressure and described similar episodic anxiety in herself. Childhood asthma was the only relevant medical history. Standardized clinical assessment ruled out schizophrenia spectrum disorders and organic brain pathology through detailed history, mental status examination, and absence of psychotic features or focal neurological signs. Screening showed moderate depressive and anxiety symptoms (Patient Health Questionnaire-9, a validated 9-item tool for assessing depression severity = 10 [16]; Generalized Anxiety Disorder-7, a 7-item scale for generalized anxiety = 11 [17]). A provisional diagnosis of obsessive-compulsive disorder with co-occurring generalized anxiety was made.

Initial pharmacotherapy consisted of low-dose escitalopram 5 mg, risperidone 0.5 mg at night, intermittent quetiapine 12.5 mg, and as-needed lorazepam 0.25-0.5 mg. Symptoms fluctuated; by mid-September, he deteriorated sharply after returning to school, describing derealization ("classmates felt like strangers") and intense anxiety (PHQ-9 = 20; GAD-7 = 18). The medication was changed to pregabalin 25-50 mg every night, risperidone 0.5-1 mg, and aripiprazole 2.5-5 mg. The scores dropped to low single digits by early 2025, but fatigue, poor focus, and short dissociative episodes continued.

Stress from exams in May-July 2025 caused a relapse: I couldn't concentrate, I couldn't sleep, and I had more "weird feelings" (including intensified health-related obsessions and derealization) (PHQ-9 = 14; GAD-7 = 12). A short lemborexant trial was ineffective. Lemborexant was selected as a newer orexin receptor antagonist targeting insomnia with a relatively favorable cognitive side-effect profile in this context [18]. After a medication holiday during a basketball camp in August, avolition and dissociation resurfaced.

Seeking faster symptom control, a glutamatergic strategy was started in September 2025: dextromethorphan (DXM) 30 mg daily, fluoxetine 10 mg to inhibit CYP2D6, and piracetam 600 mg. Fluoxetine aggravated anxiety and somatic rumination, so it was stopped after two weeks. In October-November, bupropion XL 150 mg replaced fluoxetine, DXM remained 30 mg, piracetam was raised to 1,200 mg (split dose), and L-glutamine 500 mg was added. Pregabalin 25 mg, risperidone 0.5 mg, and aripiprazole 5 mg were continued. Mood stabilized (PHQ-9 = 3-4; GAD-7 = 1-4) and daily functioning improved. By January 2026, however, bupropion seemed to provoke headaches and renewed derealization; it was discontinued (PHQ-9 = 11; GAD-7 = 11).

Due to intolerance (rather than full, adequate trials at maximum tolerated doses for 10-12 weeks), goldenseal root extract (600 mg once daily, supplying roughly 30-36 mg berberine + hydrastine) was introduced as a botanical CYP2D6 inhibitor. DXM was given as 15 mg twice daily; piracetam 600 mg twice daily was maintained. Pregabalin 25-50 mg at night, risperidone 0.5 mg, and aripiprazole 2.5 mg continued; L-glutamine and magnesium had been tried previously but were not kept long-term.

Within a month, the adolescent reported marked relief: intrusive ruminations, headaches, and somatic worries largely subsided; only mild post-lunch fatigue and occasional fleeting derealisation persisted. On January 31, 2026, he scored PHQ-9 = 2 and GAD-7 = 3, meeting remission thresholds. He attended school regularly, played basketball, and showed no functional impairment. There were no gastrointestinal problems, photosensitivity, jaundice, or any other bad effects from goldenseal during the two-month period. Laboratory monitoring was not conducted, although standard psychiatric practice includes complementary examinations (including liver function tests) for adolescents on polypharmacy with potential hepatotoxic agents, as the patient continued to be clinically asymptomatic.

**Figure 1:**
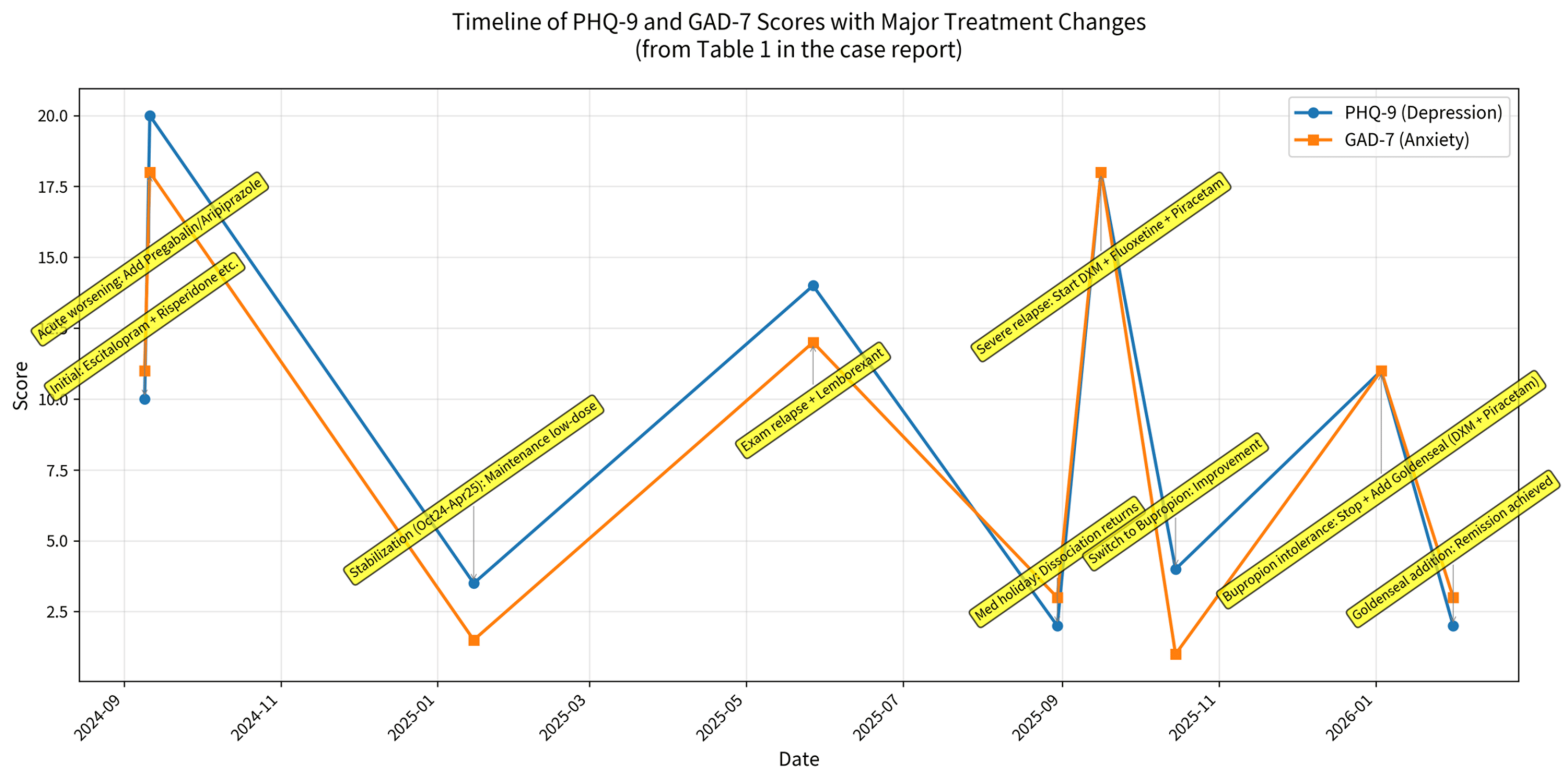
Timeline of PHQ-9 and GAD-7 scores with major treatment changes The figure displays the complete chronological data from Table 1 as a clear time-series graph: Blue line = PHQ-9 scores (depression severity); Orange line = GAD-7 scores (anxiety severity). Annotations show every major clinical event and pharmacological change (including the exact points where the glutamatergic regimen started, bupropion was replaced by goldenseal, etc.). The dramatic drop to remission thresholds (PHQ-9 = 2, GAD-7 = 3) after goldenseal addition on January 3, 2026, is clearly visible. The x-axis uses real calendar dates; the midpoint for range periods (Oct 2024-Apr 2025 and Oct-Nov 2025) is used for accurate placement. Abbreviations: PHQ-9 = Patient Health Questionnaire-9; GAD-7 = Generalized Anxiety Disorder-7

This course (Table 1; Figure 1) illustrates that an oral glutamatergic stack--DXM, an effective CYP2D6 inhibitor, and piracetam--can be adapted for a treatment-resistant adolescent with OCD. When conventional inhibitors were not tolerated, goldenseal provided sufficient metabolic inhibition to sustain DXM exposure, contributing to sustained remission without evident adverse effects.

**Table 1:** Chronological clinical course, symptom scores, and pharmacological management Abbreviations: PHQ-9 = Patient Health Questionnaire-9; GAD-7 = Generalized Anxiety Disorder-7; OCD = Obsessive-Compulsive Disorder; GAD = Generalized Anxiety Disorder; PRN = As needed; BID = Twice daily; CYP2D6 = Cytochrome P450 2D6.

Date	Scores (PHQ-9 / GAD-7)	Clinical Presentation & Symptoms	Pharmacological Regimen (Daily Dosing)
Sep 09, 2024	10 / 11	Initial Presentation: Health anxiety, intrusive thoughts regarding past head injuries, avolition, poor sleep. Provisional diagnosis of OCD with GAD.	Initiation: Escitalopram 5 mg; Risperidone 0.5 mg; Quetiapine 12.5 mg (intermittent); Lorazepam 0.25–0.5 mg (PRN)
Sep 11, 2024	20 / 18	Acute Deterioration: Severe worsening upon return to school. Reported derealisation ("classmates felt like strangers") and intense anxiety.	Adjustment: Pregabalin 25-50 mg (added); Risperidone 0.5-1 mg (titrated); Aripiprazole 2.5-5 mg (added); Escitalopram discontinued
Oct 2024 – Apr 2025	Range: 3–4 / 0–3	Stabilization: Mood and anxiety scores dropped to remission levels. Functioning improved; engaged in basketball and schoolwork.	Maintenance: Pregabalin 25-50 mg; Risperidone 0.5-1 mg; Aripiprazole 2.5-5 mg
May 27, 2025	14 / 12	Relapse (Exam Stress): Recurrence of "weird feelings," poor concentration, and insomnia precipitated by academic pressure.	Adjustment: Lemborexant 2.5-5 mg (added for sleep); Risperidone 0.5 mg; Aripiprazole 2.5 mg
Aug 30, 2025	2 / 3 (Pre-relapse)	Medication Holiday: Patient stopped medications during a trip/camp. Reported "brain felt blank" and return of dissociation.	Medications were halted by the patient.
Sep 16, 2025	18 / 18	Severe Relapse: Intrusive thoughts (OCD-like), nightmares, avolition, and derealisation.	Glutamatergic Strategy Initiated: Dextromethorphan (DXM) 30 mg; Fluoxetine 10 mg (as CYP2D6 inhibitor); Piracetam 600 mg
Oct – Nov 2025	4 / 1	Intolerance to Fluoxetine: Fluoxetine aggravated anxiety/somatic rumination (stopped after 2 weeks). Improvement on Bupropion: Mood stabilized, functioning improved.	Regimen Change: DXM 30 mg; Bupropion XL 150 mg (replaced Fluoxetine); Piracetam 1,200 mg; L-Glutamine 500 mg (added); Antipsychotics/Pregabalin continued
Jan 03, 2026	11 / 11	Intolerance to Bupropion: Worsening somatic symptoms (headache) and renewed derealisation attributed to Bupropion.	Discontinuation: Bupropion stopped. Botanical strategy initiated: Goldenseal root 600 mg (added as CYP2D6 inhibitor); DXM 30 mg (15 mg BID); Piracetam 1,200 mg (600 mg BID)
Jan 31, 2026	2 / 3	Remission: Marked relief of intrusive ruminations and headaches. Mild residual fatigue. Full return to school and sports. No adverse effects from the botanical agent.	Current Regimen: DXM 30 mg (15 mg BID); Goldenseal Root 600 mg; Piracetam 1,200 mg (600 mg BID); Pregabalin 25-50 mg; Risperidone 0.5 mg; Aripiprazole 2.5 mg

Ethics

Written informed consent was obtained from the patient and his legal guardian for both the off-label glutamatergic regimen and the publication of this case report, with all identifying details anonymized.

## Discussion

The present report documents marked and sustained clinical improvement in a 16-year-old with treatment-intolerant OCD after the introduction of an oral glutamatergic stack: dextromethorphan for rapid NMDA antagonism, piracetam for AMPA facilitation, and goldenseal root to slow dextromethorphan metabolism by moderating CYP2D6 activity. Two conventional enzyme blockers, fluoxetine and later bupropion, were abandoned because each intensified anxiety or dissociative complaints. Replacing them with a botanical alternative proved decisive; symptom scores fell quickly, and functional recovery followed.

Mechanistic rationale arises from increasing evidence that transitioning cortical circuits from "NMDA dominant" to "AMPA dominant" can yield swift alleviation in mood and anxiety spectra [13,19]. Intravenous ketamine can make this change, but it is hard to do because of dissociation, cost, and how the drug is given. Auvelity, a fixed tablet of dextromethorphan plus bupropion, partly reproduces ketamine's speed in major depression, yet its benefit is less robust, likely because it provides NMDA blockade without an explicit AMPA boost [10]. Cheung's four-component concept, therefore, adds piracetam and glutamine to complete the plasticity cascade. In the current case, glutamine was discontinued without a clear effect; nevertheless, the two remaining pharmacodynamic "pushes" plus enzymatic protection were sufficient for clinical remission.

In a recent polygenic study of more than 68,000 cases and controls, variants clustered most strongly in gene sets that orchestrate complement-driven microglial trimming, showing up to 1.32-fold heritability enrichment with corrected p values dipping below 10⁻¹⁰³; in contrast, glutamatergic genes were essentially silent [20]. If OCD starts with aggressive developmental pruning that makes cortico-striato-thalamo-cortical loops unstable, then drugs that rebuild or strengthen surviving synapses may help fix the problem. Both dextromethorphan and piracetam are known to boost AMPA throughput, stimulate brain-derived neurotrophic factor release, and engage mTOR signaling, a constellation that supports new spine formation and circuit rewiring. The present case, therefore, offers clinical support for the pruning model, suggesting that readily available, non-serotonergic compounds can restore synaptic density and relieve obsessive-compulsive symptoms in a manner conceptually similar to ketamine but far easier to deliver.

Goldenseal supplies berberine and hydrastine, alkaloids that reduce CYP2D6 activity by roughly one-half in healthy volunteers [14]. Goldenseal is an herbal supplement with demonstrated CYP2D6 inhibitory effects in phenotyping studies, but its clinical use in psychiatry lacks robust validation. It should be regarded as a botanical with variable alkaloid content and potential risks (including hepatotoxicity with prolonged use and interactions with CYP-metabolized antipsychotics). At 600 mg daily, the patient tolerated the extract without hepatic or gastrointestinal complaints across two months, mirroring the short-term safety profile seen in controlled phenotyping trials [21]. The rapid drop in PHQ-9 from 11 to 2, alongside the disappearance of intrusive ruminations, suggests that moderate inhibition was pharmacologically meaningful. Because plasma levels were not measured, the inference remains indirect. We acknowledge the absence of plasma monitoring and routine liver function tests (AST/ALT); the decision was based on short-term use (two months), clinical stability, and lack of suggestive symptoms. Potential interactions with ongoing low-dose risperidone and aripiprazole were monitored clinically with no observed adverse effects.

Transcranial magnetic stimulation (TMS) represents a low-risk non-pharmacological alternative for treatment-intolerant cases, though it was not pursued here due to limited local availability and economic constraints [3].

Low-dose risperidone and aripiprazole remained in place throughout and may have offered ancillary anti-obsessional benefit, consistent with meta-analytic data for antipsychotic augmentation in youth OCD [5]. Pregabalin probably helped with residual somatic anxiety. But these drugs had been present during previous relapses, which shows how different the glutamatergic stack is.

This finding is consistent with small adult studies indicating that dextromethorphan-based combinations can alleviate refractory OCD [12], but it expands the notion to include adolescents and a natural CYP2D6 inhibitor. Using an over-the-counter herbal remedy can be helpful when prescription inhibitors are hard to get or don't work well; however, the fact that alkaloid levels vary, there is no standardization, and there are signs of liver damage with long-term use [15] means that you need to be careful. Until high-quality controlled data become available, it is wise to do routine lab tests and get supplies from trusted sources.

Limitations encompass the single-case design, lack of objective pharmacokinetic validation, absence of laboratory safety monitoring for goldenseal, and the presence of concurrent psychotropics that obscure attribution. Placebo effects, maturation, or regression to the mean cannot be excluded. A longer follow-up will clarify durability and safety. Ethical considerations for botanical supplements in adolescents include careful risk-benefit assessment and informed consent, especially without laboratory monitoring.

## Conclusions

This case suggests that an adolescent with treatment-intolerant OCD experienced marked improvement after a glutamatergic regimen combining dextromethorphan, piracetam, and goldenseal-mediated CYP2D6 inhibition. The result supports systematic investigation of affordable, orally administered plasticity-based therapies in youth anxiety-obsessional disorders, with particular attention to pharmacokinetics, botanical product quality, and long-term safety.
